# Metal-Based Approaches for the Fight against Antimicrobial Resistance: Mechanisms, Opportunities, and Challenges

**DOI:** 10.1021/jacs.4c16035

**Published:** 2025-03-10

**Authors:** Chenyuan Wang, Xueying Wei, Liang Zhong, Chun-Lung Chan, Hongyan Li, Hongzhe Sun

**Affiliations:** † Department of Chemistry, The University of Hong Kong, Pokfulam Road, Hong Kong SAR, PR China; ‡ CAS-HKU Joint Laboratory of Metallomics for Health and Environment, The University of Hong Kong, Pokfulam Road, Hong Kong SAR, PR China; § Department of Microbiology, The University of Hong Kong, Pokfulam Road, Hong Kong SAR, PR China; ∥ State Key Laboratory of Synthetic Chemistry, The University of Hong Kong, Pokfulam Road, Hong Kong SAR, PR China

## Abstract

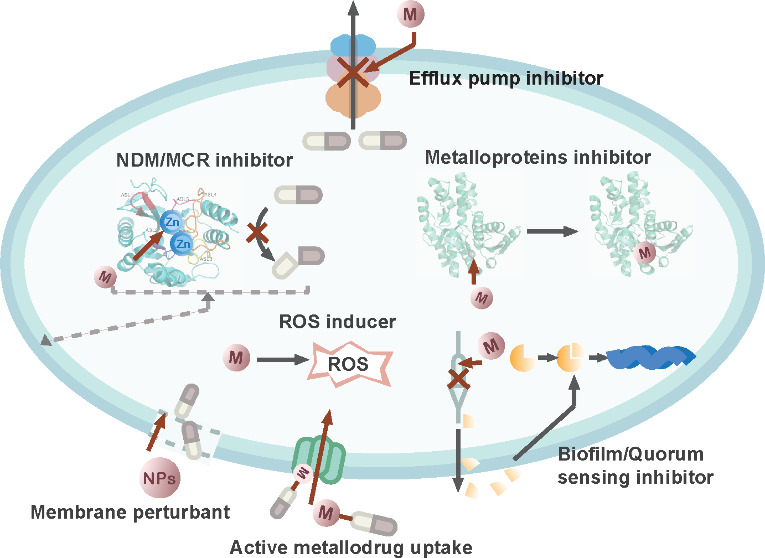

The rapid emergency and spread of antimicrobial-resistant (AMR) bacteria and the lack of new antibiotics being developed pose serious threats to the global healthcare system. Therefore, the development of more effective therapies to overcome AMR is highly desirable. Metal ions have a long history of serving as antimicrobial agents, and metal-based compounds are now attracting more interest from scientific communities in the fight against AMR owing to their unique mechanism. Moreover, they may also serve as antibiotic adjuvants to enhance the efficacy of clinically used antibiotics. In this perspective, we highlight important showcase studies in the last 10 years on the development of metal-based strategies to overcome the AMR crisis. Specifically, we categorize these metallo-antimicrobials into five classes based on their modes of action (i.e., metallo-enzymes and metal-binding enzyme inhibitors, membrane perturbants, uptake/efflux system inhibitors/regulators, persisters inhibitors, and oxidative stress inducers). The significant advantages of metallo-antimicrobials over traditional antibiotics lie in their multitargeted mechanisms, which render less likelihood to generate resistance. However, we notice that such modes of action of metallo-antimicrobials may also raise concern over their potential side effects owing to the low selectivity toward pathogens and host, which appears to be the biggest obstacle for downstream translational research. We anticipate that combination therapy through repurposing (metallo)drugs with antibiotics and the optimization of their absorption route through formulation to achieve a target-oriented delivery will be a powerful way to combat AMR. Despite significant challenges, metallo-antimicrobials hold great opportunities for the therapeutic intervention of infection by resistant bacteria.

## Introduction

Antimicrobial resistance (AMR), a silent pandemic, has emerged as a significant public health concern worldwide. AMR was listed as one of the top 10 global health threats, leading to the loss of 4.95 million lives in 2019. According to the World Health Organization (WHO), the diseases associated with drug resistance claim at least 700,000 lives annually, with 1.6 million succumbing to tuberculosis caused by bacteria that are resistant to most first-line medications.^1^ The rise of infections caused by antimicrobial-resistant pathogens has rapidly decreased the armamentarium of effective antibiotics. If no action is taken, it is estimated that AMR could cost the world’s economy USD 100 trillion by 2050 according to the WHO. The arduous process of novel antibiotic discovery substantially trails the development of drug resistance in microorganisms, which highlights the limitation of the current design of antibiotics. Instead of specifically targeting the essential physiological or metabolic functions of the bacterial cell, a pressing need is to develop antimicrobial agents with different mechanisms of action such as a multitargeted mode of action, which will slow down the occurrence of resistant bacterial strains.

The use of metals as antimicrobial agents stretches back from ancient time to the early 20th century, only fading out until the introduction of organic antibiotics in the mid-20th century. Recently, metallo-antimicrobials have been regaining attention and are considered to be one of the promising solutions to combat the current AMR crisis due to their unique 3D geometry and physical properties^2^ and multiple mechanisms of actions.^3^ In contrast to the “magic-bullet” concept utilized in the development of conventional antibiotics by primarily targeting specific biochemical processes, which in turn provide the ease of developing progressive resistance for bacteria, metallo-antimicrobials appear to target multiple cellular processes, leading to pleiotropic effects on bacterial cells.^4^ Metallo-antimicrobials are extensively used in clinics for the treatment of various diseases through multiple mechanisms such as covalent binding to biomolecules, redox activity, photoactivity, and radioactivity.^5^ Thus, renewed interest resides in the potentials that the metallo-antimicrobials hold as either antimicrobials or antibiotic adjuvants, and such metal-based strategies provide an effective alternative to conventional antibiotics not only to kill AMR pathogens but also to have less likelihood to develop resistance.

**Figure 1:**
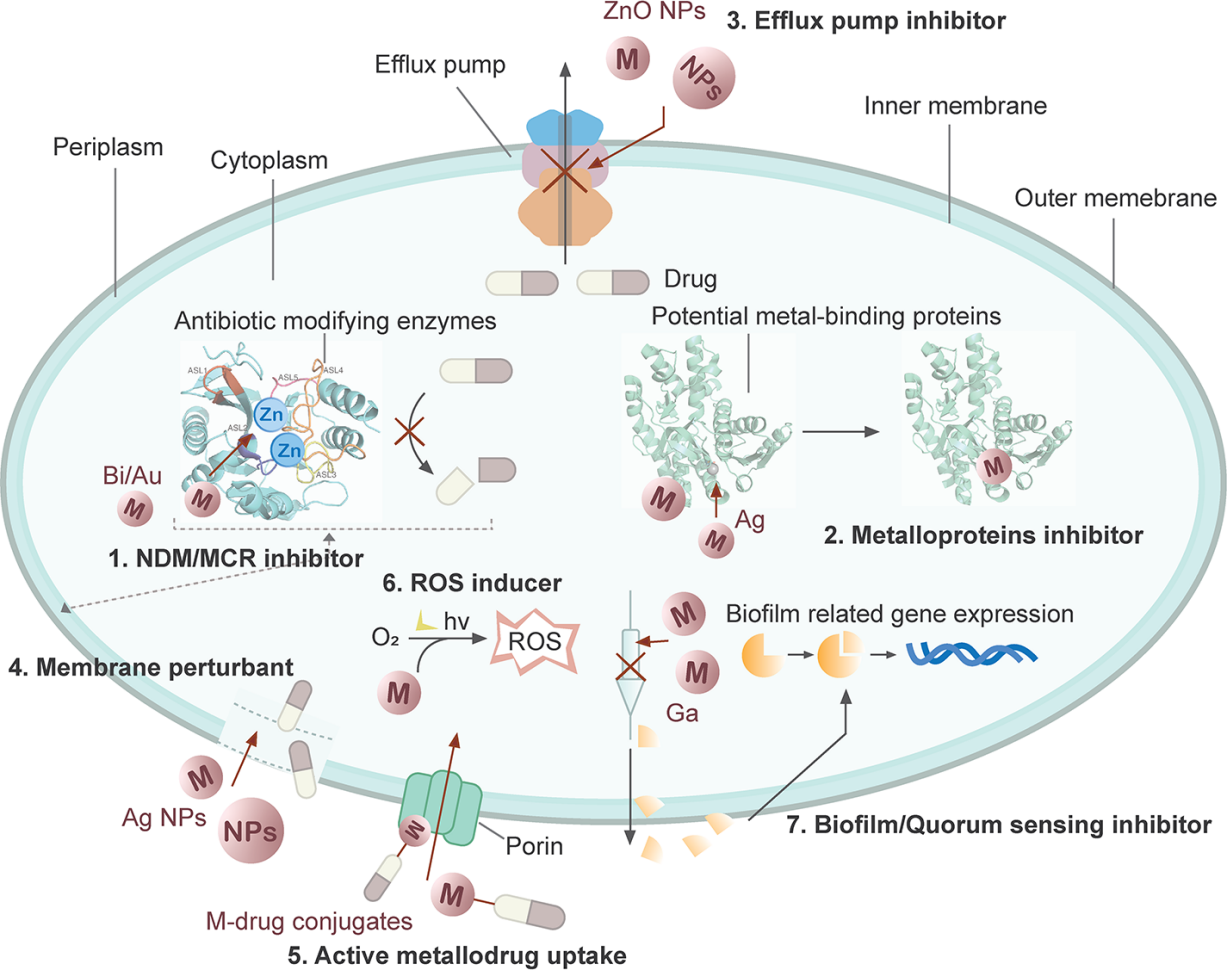
Overview of metal-based antimicrobial agents in combating antimicrobial resistance (AMR). These metallo-antimicrobial agents can function as (1) suppressors of New Delhi metallo-β-lactamase (NDM) and mobilized colistin resistance (MCR); (2) inhibitors of metalloproteins; (3) inhibitors of efflux pumps; (4) disruptors of bacterial cell membranes; (5) active metallo-drug uptake; (6) inducers of reactive oxygen species (ROS); and (7) inhibitors of biofilm formation and quorum sensing.

In this perspective, we focus on the transition metal and some IIIA and VA group metals, highlight the most updated progress in the last 10 years of the development of metallo-antimicrobials (e.g., metal compounds/complexes, alloys, organometallics, metal nanoparticles, and metal–drug conjugates) in combating AMR both as resistance breakers and antimicrobials, and classify these metallo-antimicrobials by five main modes of actions: (a) metallo-enzyme and metal-binding enzyme inhibitors, (b) membrane perturbants, (c) uptake/efflux system inhibitors/regulators, (d) persisters inhibitors, and (e) oxidative stress inducers (Figure 1). A majority of metallo-antimicrobials exhibit activity through multiple mechanisms, providing them the flexibility and tolerance to combat single-mechanism-related AMR. We also enumerate the opportunities and challenges in this field.

## Metallo-agents as Resistant Enzyme Inhibitors

### β-Lactamase Inhibitors

**Figure 2:**
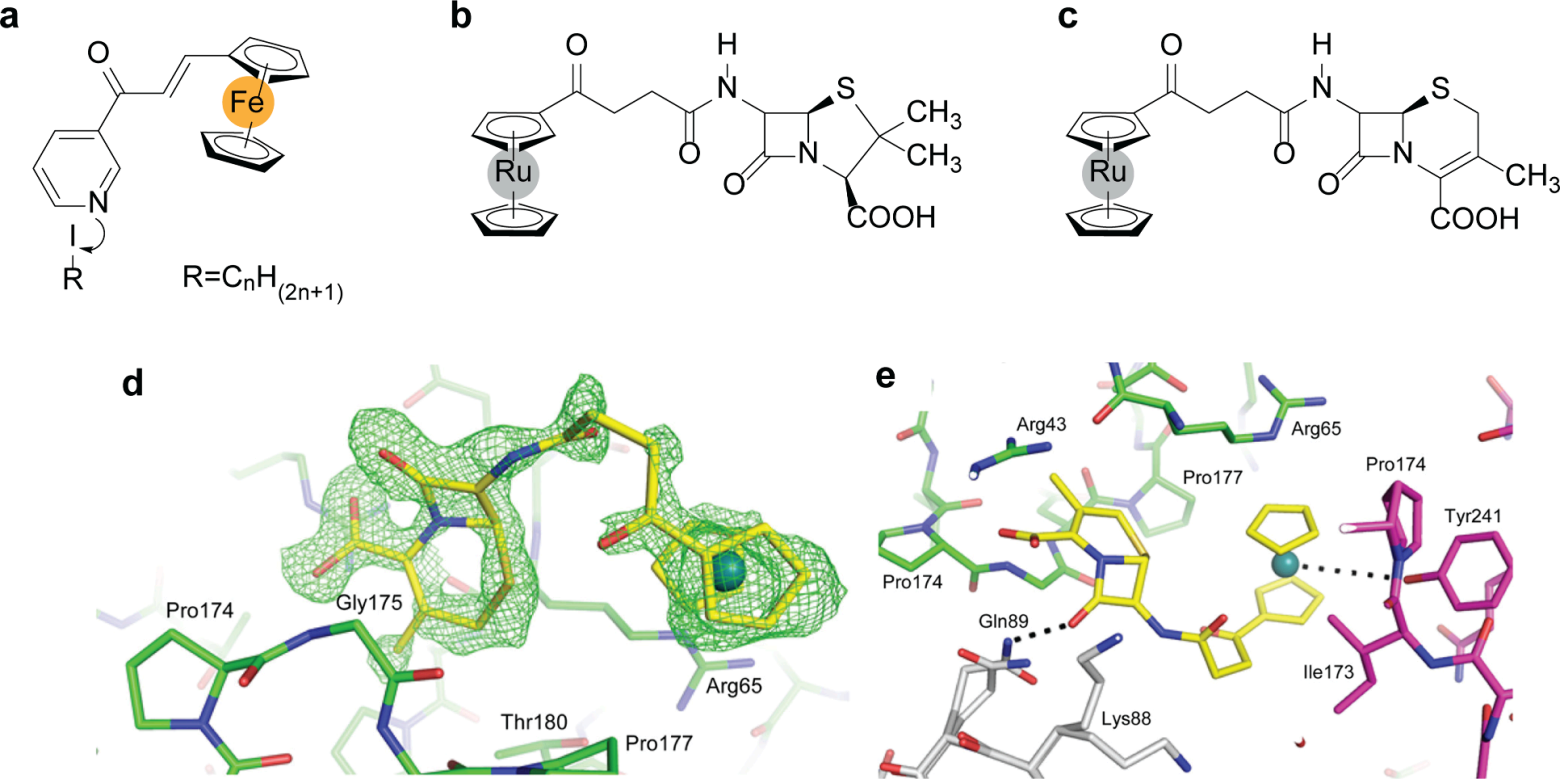
Chemical structure of (a) ferrocenyl chalcones derivatives, (b) the ruthenocenyle-6-aminopenicillanic acid (6-APA) conjugate, and (c) metallo-cenyl-7-ADCA3. (d) Intact-7-ADCA3 (yellow) observed at the CTX-M β-lactamase crystal-packing interface: unbiased Fo–Fc density map is shown in green at 3σ. (e) Interactions between compound 3 and three protein monomers. Monomers 1–3 are colored in green, white, and magenta, respectively. Potential hydrogen bonds are shown as black dashed lines. Adapted from ref (14). Copyright 2017, American Chemical Society.

β-Lactams, including penicillin, cephalosporins, carbapenems, and monobactams, are a class of antibiotics that contain a β-lactam ring and are widely used in the treatment of various bacterial infections. These antibiotics work by binding to PBPs (penicillin-binding proteins) and inhibiting the synthesis of bacterial cell walls, leading to bacterial cell death. However, bacteria have developed resistance to β-lactam antibiotics through various mechanisms, including the production of β-lactamases enzymes, which are capable of hydrolyzing and inactivating β-lactam antibiotics.^6^ β-Lactamases can be classified into four distinct classes, namely, A, B, C and D, based on specific sequence motifs and hydrolytic mechanisms.^7^ Targeting β-lactamases to inhibit the hydrolyzation of the antibiotic has been a promising strategy for combating AMR.^8^ Ferrocene and its derivatives were reported as active agents against parasitic,^9^ bacterial, and fungal infections for several years.^10,11^ Recently, a series of ferrocenyl chalcone derivatives were reported (Figure 2a) as antibacterial agents and could inhibit clinically isolated methicillin-resistant *Staphylococcus aureus* (MRSA) strains with MIC (Minimum inhibitory concentration) values of 0.008–0.063 mg/mL (∼10–100 μM).^12^ Metallocene derivatives usually functioned as serine β-lactamase inhibitors that protect antibiotics from hydrolyzation. For example, a novel ruthenocenyle-6-aminopenicillanic acid (6-APA) conjugate (Figure 2b) exhibited high potency against *S. aureus* with an MIC of 4 μg/mL.^13^ The enhancement of antibacterial activity comes from the noncovalent binding of the ruthenocentyle to the active site of the serine β-lactamase CTX-M. Metallocenyl-7-ADCA3 (Figure 2c) was also reported as a CTX-M β-lactamase inhibitor by noncovalent binding to protect the β-lactam ring from cleavage.^14^ X-ray structure (Figure 2d,e) revealed that the compound binds to the residues of Asn104, Ser130, Asn132, Thr235, and Ser237. Ferrocene derivatives as serine β-lactamase inhibitors have provided a new scaffold molecule for combating β-lactamase and restoring the antibacterial activity of the β-lactams. One advantage of ferrocene derivatives is their ability to diffuse across the cell membrane due to their high lipophilicity.

**Figure 3:**
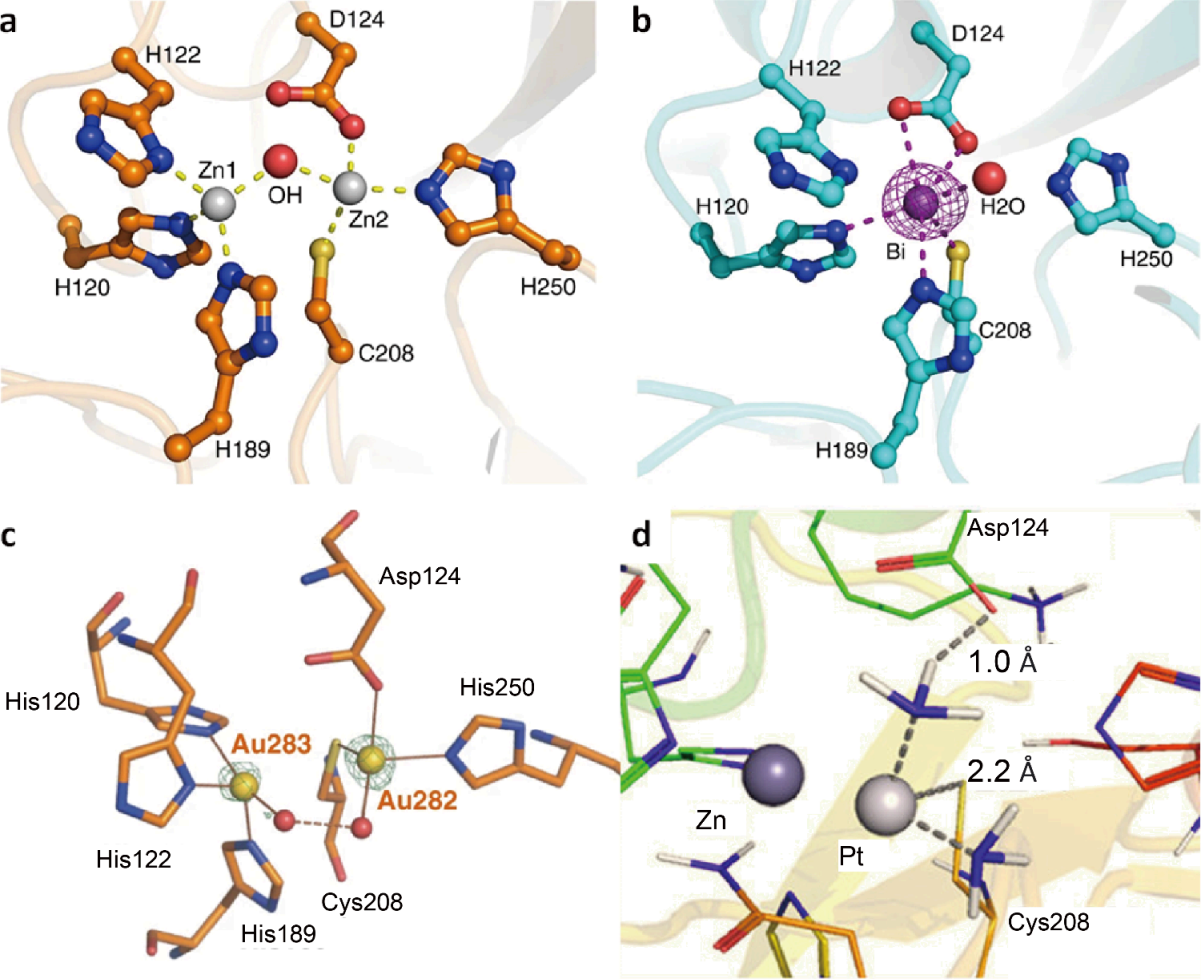
Crystallographic analysis of the binding mode of (a) native Zn-NDM-1, (b) Bi-NDM-1, and (c) Au-NDM-1. (d) Molecular docking of the binding mode of Pt(II) with NDM-1. Adapted from ref (23) (available under a CC-BY 4.0 license; copyright 2018, The Author(s)) and ref (25) (copyright 2020, American Chemical Society).

**Figure 4:**
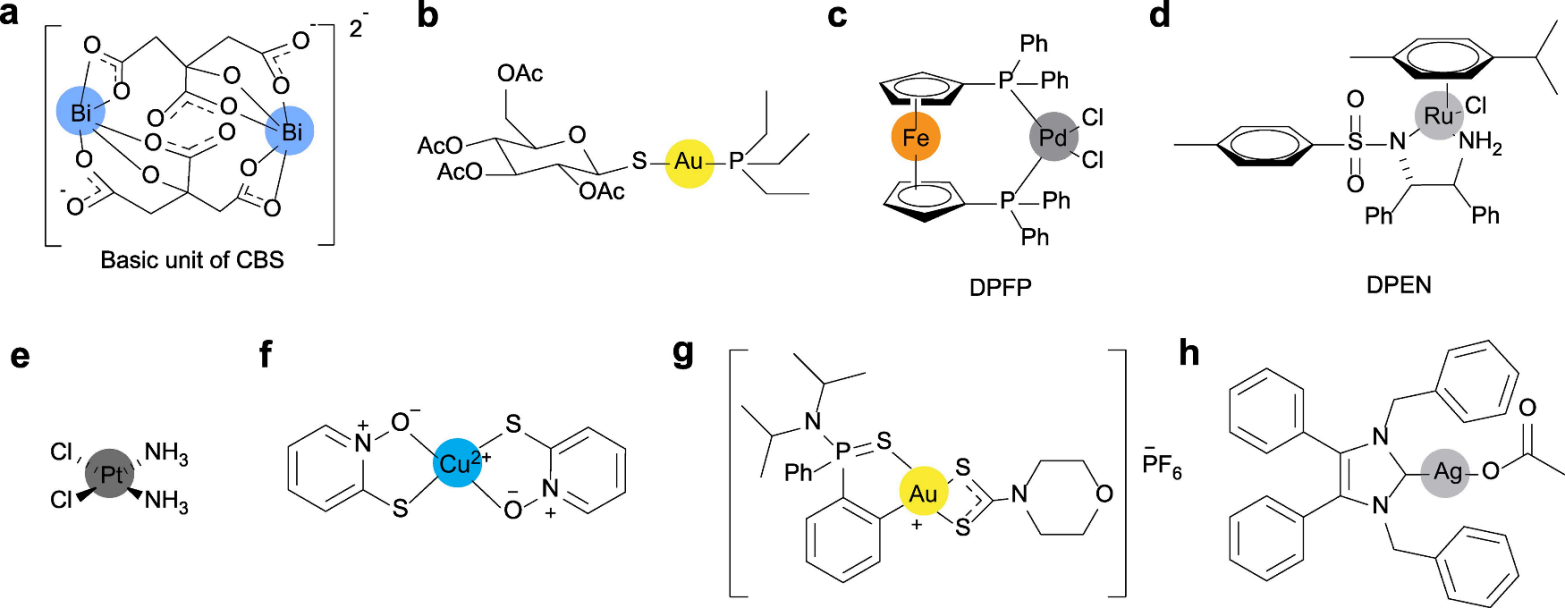
Chemical structures of (a) colloidal bismuth citrate (CBS), (b) auranofin, (c) cisplatin, (d) Pd(II) complex DPFP, (e) Ru(III) complex DPEN, (f) copper-pyrithione, (g) (C∧S)-cyclometalated Au(III) dithiocarbamate complex, and (h) N-heterocyclic carbene.

Metallo-β-lactamases, or MBLs classified as group B, are a heterogeneous group of zinc metallo-enzymes which utilize a metal-activated water nucleophile to catalyze the hydrolytic reaction of the β-lactam ring. Owing to their ability to hydrolyze virtually all β-lactam antibiotics and the lack of clinically effective MBL inhibitors, MBLs are becoming a topic of significant interest and concern.^15^ In particular, the New Delhi metallo-β-lactamase-1 (NDM-1), which was first identified in 2009, has rapidly disseminated more than 70% worldwide.^16^ Its emergence and spread pose a significant threat to public health, as NDM-1 has the capacity to hydrolyze carbapenems, which are considered to be the last line of defense against severe bacterial infections.^17^ The active center of NDM-1 consists of two positively charged Zn(II) ions, which are responsible for the hydrolysis by enhancing the nucleophilicity to attack the β-lactam ring (Figure 3a).^18,19^ Zn1 is coordinated to His120, His122, and His189 whereas Zn2 is coordinated to Asp124, Cys208, and His250. Consequently, the development of NDM-1 inhibitors primarily centers around interference with the essential zinc coordination and thus inactivation of the enzyme. Enormous effort has been made in the development of MBL inhibitors,^20^ for which the Zn(II) ions are either kicked out as in the case of aspergillomarasmine A (AMA)^21^ or complexed by agents such as VNRX-5133.^22^ Alternatively, the cofactor zinc could be replaced by metallo-drugs and relevant compounds such as Bi(III),^23^ Au(I),^24^ and Pt(II)^25^ (Figure 4a,b,e), resulting in irreversible inhibition of the enzymes.

Considering that NDM-1 relies on the zinc cofactors for its enzymatic activity, metal compounds with coordination properties similar to those of zinc ions could be potential inhibitors of NDM-1. Indeed, the FDA-approved drug for the treatment of peptic ulcers for decades,^26^ colloidal bismuth citrate (CBS), was demonstrated to be a potent NDM-1 inhibitor.^23^ CBS could inhibit NDM-1 and other MBLs with IC_50_ values of 0.70–3.55 μM *in vitro*. CBS and other Bi(III) compounds could resensitize MBL-positive *E. coli* as well as other bacterial strains to Meropenem both *in vitro* and in the murine infectious model. Different from other small-molecule MBL inhibitors, Bi(III) exhibits broad-spectrum inhibition through irreversible displacement of the zinc cofactors. One Bi(III) displaces two Zn(II) ions and coordinates with Cys208 at the active site, as revealed from the X-ray structure (Figure 3b). Similarly, auranofin, the Au(I)-based antirheumatic drug, exhibited potent inhibition toward NDM-1.^24^ The crystal structures showed auranofin irreversibly formed Au-NDM-1 complexes by displacing Zn(II) ions in the active sites (Figure 3c). The synergistic effect between auranofin and the β-lactam antibiotic was also demonstrated both *in vitro* and in murine infection models. Besides Bi(III) and Au(I), cisplatin and palladium(II) complexes (Figure 4c) were also reported as potent MBL inhibitors against carbapenem-resistant *Enterobacteriaceae* strains by displacement of Zn(II).^25^ Cisplatin showed IC_50_ ranging from 0.14 to 7.60 μM against NDM-1 and other MBLs and could improve Meropenem activity by 2–64 times against *E. coli* (NDM-1^+^) strains and other Gram-negative bacterial (NDM-1^+^) strains. Based on molecular docking, Pd(II) was found to bind to the Zn2 site in NDM-1 with a binding energy of −5.96 kcal/mol, whereas cysteine residue Cys208 binds Pt(II) and amine ligands of cisplatin bind to aspartic acid (Figure 3d). However, compared with Bi(III) and Au(I) compounds, cisplatin and Pd(II) demonstrated severe cell toxicities, which needs to be further examined. Furthermore, Ru complexes also exhibited broad-spectrum inhibitory activity against MBLs.^27^ The binding of DPEN (Figure 4d) to the active site of NDM-1 affects the hydrophobic pocket and displaces Zn(II) at the active site, specifically at the Cys208 and Met67 residues with IC_50_ of 0.46 ± 0.24 μM. Moreover, Cu(II) (Figure 4f) has been reported to be able to potentiate the activity of carbapenems against NDM-1^+^*E. coli* with an FIC index value of 0.11 for ertapenem and Meropenem,^28^ which is attributable to the capability of Cu(II) directly inactivating the NDM-1 activity *in vitro*, and synergistically restored the antibacterial activity of Meropenem. Cu(II) could either bind to the zinc site, displacing the zinc ions, or bind to an allosteric site through a noncompetitive mode. Taken together, we believe that clinically used metallo-drugs hold great potential to be repurposed to serve as antibiotic adjuvants in the fight against AMR.

The development of metal-based MBL inhibitors via the replacement of the Zn(II) cofactors by other metal ions opens up new horizons for the clinical treatment of AMR. According to the general HSAB (hard–soft-acid–base) features, Bi(III), Au(I), Pt(II), Pd(II), Ru(III), and Cu(II) are considered to exhibit similar coordinating properties to Zn(II), which has a high affinity for the thiolate of cysteine and the imidazole of histidine, both of which exist in the MBL active site. Therefore, these metal-containing compounds can potentially serve as effective inhibitors of MBLs for the fight against AMR. However, the potential side effects may occur due to the lack of selectivity of the binding between metal ions and nucleic acids. We anticipate that the repositioning of clinically used metallo-drugs, e.g., CBS and auranofin, might be a short-term solution, given the well-documented safety profiles of these drugs. However, as CBS is used for local infection, it may not be suitable for systematic infection, in particular, through oral administration. Alternative administration routes such as pulmonary delivery might be an option to resolve the low bioavailability issue. It remains to be a challenge to develop more potent metal-based MBL inhibitors with improved selectivity, thus reducing the potential side effects of these inhibitors.

#### MCR-1 Inhibitors

**Figure 5:**
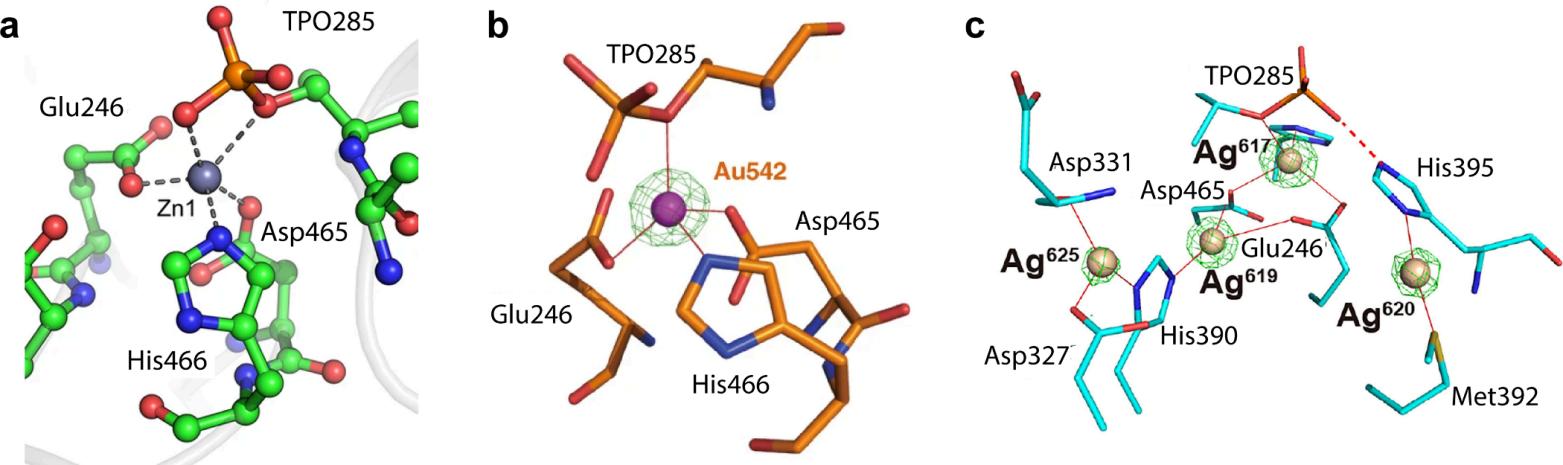
X-ray crystal structures of (a) Zn(II)-bound (i.e., Zn-MCR-1) (PDBID: 5LRN), (b) Au(I)-bound (Au-MCR-1) (PDBID: 6LHE), and (c) Ag(I)-bound MCR-1 (PDBID: 7WAA). Note that Au(I) occupies the Zn1 site, and a tetra-Ag(I) center is present in the protein. Partial figure is adapted from ref (24) (available under a CC-BY 4.0 license; copyright 2020, the author(s)), ref (33) (available under a CC-BY 4.0 license; copyright 2017, the author(s)), and ref (37) (available under a CC-BY 4.0 license; copyright 2022, the author(s)).

mcr-1 is a plasmid-mediated colistin resistance gene that encodes for phosphoethanolamine (pEtN) transferase known as MCR-1, which was first found in Gram-negative bacteria in 2015 and now has been disseminated over 40 countries.^29^ MCR-1 modifies lipid A of lipopolysaccharide of the bacterial cell membrane, reducing the electrostatic attraction to colistin, a last-resort antibiotic, and thereby conferring resistance.^30^ Due to the emergence of the plasmid-borne transmissible mcr-1 gene, the spread of MCR-1 is of significant concern, and the efficiency of colistin was further challenged. In addition, the *mcr-1* gene could cotransfer with other *mcr* genes and even with the *bla*_MBL_ gene encoding NDM-1.^31,32^ In the active site of MCR-1, a zinc ion coordinates with Glu246, Asp465, His466, and phosphorylated Thr285 (TPO285), which is essential for its function (Figure 5a).^33^ Variable numbers of Zn(II) ranging from 1 to 4 were observed with the active site in different crystal structures.^34^

Similar to NDM-1, Zn(II) at the active site of MCR-1 is a prime target for the development of inhibitors, given the crucial role it plays during the biological process. Followed by the zinc displacement strategy, we found that auranofin is an effective dual inhibitor of both NDM-1 and MCR-1 with IC_50_ of 437.9 ± 29.1 nM.^24^ Zinc ions in the active site of MCR-1 were displaced irreversibly by Au(I) from auranofin, and Au(I) was shown to bind Glu246, Asp465, His466, and TPO285 as revealed by X-ray crystallography (Figure 5b). Consequently, the function of MCR-1 was disrupted, and the susceptibility of colistin-resistant bacteria was restored. The combination therapy consisting of auranofin and colistin exhibited antibacterial ability against MCR-1 positive bacteria both *in vitro* and in the peritonitis infection animal model. In addition to auranofin, other gold-based drugs and nanoparticles also exhibited synergy with colistin against MCR-1 positive bacteria via the zinc displacement mechanism.^35^ The key factor in the degrees of synergism is attributed to the release of Au(I) from different ligands. Besides gold compounds, Ag(I), including silver nanoparticles, could also restore colistin efficacy against MRSA by inhibiting the activity of the MCR-1 enzyme. Silver has a long history of being used for its antibacterial properties, dating back centuries. Silver compounds, such as silver sulfadiazine, have been utilized in various forms to combat infections and promote healing, particularly in the treatment of severe burns.^36^ We have found that Ag(I) restored the antibacterial activity of colistin against *mcr* carrying multi-drug-resistant *S. aureus* with a FIC index value of 0.375.^37^ A tetra-Ag(I) center within the active-site pocket of MCR-1 was formed through the displacement of zinc ions at the active site, leading to enzyme inactivation (Figure 5c).

Given that the coexpression of MCR-1 and MBLs would occur in multi-drug-resistant bacteria cells, the development of an inhibitor capable of targeting both enzymes simultaneously becomes crucial. However, it poses challenges for organic inhibitors due to the substantial structure differences in the active sites and modes of action between the two resistant genes (i.e., *mbl* and *mcr*). However, metallo-antimicrobials, such as auranofin (in phase I clinical trial as antiparasitic agent),^183^ exhibit promising dual inhibition against MBLs and MCRs via the metal displacement mechanism, offering a potent arsenal in the fight against microbial resistance caused by multiple-resistant genes. Further optimization of gold-based compounds with less toxicity may have great potential for clinical application.

##### TrxR Inhibitors

Metalloproteins play key roles in various biological processes, including respiration and photosynthesis. Metal–protein interactions also play vital roles for metal-based drug metabolism and were considered to be potential novel targets for metal-based drugs.^38^ The exogenous metal ions could coordinate to metallo-proteins at the metal centers, resulting in the loss of function of the metallo-proteins. Alternatively, the exogenous metal ions could also bind to metallo-proteins allosterically, leading to structural changes and thus disrupting the functions of metallo-proteins. Moreover, certain protein functions could be disrupted upon binding of exogenous metal ions or metallo-drugs. For example, thioredoxin reductase (TrxR) from the Gram-positive bacterial antioxidant thioredoxin system (TS) with a conserved active site sequence of Cys-Pro-Gly-Cys was suggested as a novel antimicrobial target in Gram-positive and some Gram-negative bacteria.^39^ The antioxidant thioredoxin system plays a crucial role in many physiological processes, ranging from the reduction of nucleotides to the detoxification of xenobiotics, oxidants, and radicals.^40^ The reduction of disulfide bonds is mediated by two major enzymes, thioredoxin and glutaredoxin, both of which have a motif of Cys-X1-X2-Cys in the active site. In most prokaryotic and eukaryotic cells, reduction can be mediated in parallel by two major thiol-dependent systems, the Trx-TrxR system and glutathione reductase GSH-GR system, which are responsible for the electron transfer from NADPH.^41^ These two antioxidant systems are crucial for many biological processes including DNA synthesis, defensive oxidative stress, and post-translational modifications. The glutaredoxin system is lacking in many Gram-positive bacteria such as *S. aureus*, thus the Trx-TrxR system is more essential in these organisms and the disruption of thioredoxin reductase (TrxR) triggers the accumulation of cellular oxidants and inhibits the bacterial growth, making it a potential drug target for treating bacterial infections.

Metallo-antimicrobials with high thiol reactivity and high binding affinity toward cysteine residues, for example, auranofin, are considered to be an inhibitor of cysteine-containing enzymes. Auranofin (Figure 4b) was first identified as a broad-spectrum TrxR inhibitor in 2015.^42^ It was reported to inhibit *M. tuberculosis* and *S. aureus* TrxR *in vitro* with MIC values of 0.4–14.7 μM in a dose-dependent manner via the formation of the Au(I)-Cys complex at the active site of TrxR. The crystal structure of the *Eh*TrxR (TrxR from *Entamoeba histolytica*) dimer reveals that Au(I) binds to the nonconserved Cys286 instead of the Cys140-Cys143.^43^ The efficacy of auranofin was further examined by screening >500 clinical *S. aureus* isolates, and an Au(I)-TrxR complex structure was proposed that Au(I) coordinates to the catalytic cysteine pair, leading to inhibition of the enzyme activity, although these cysteines form a disulfide bond.^44^ Besides auranofin, the Au(III) complex was also synthesized to be a potential TrxR inhibitor recently.^45^ Au(III)-dithiocarbamate (dtc) complex (Figure 4g) exhibited antibacterial activity against Gram-positive bacteria, including MRSA, *S. epidemidis*, and *S. pneumpniae* strains with MICs ranging from 0.15 to 2.44 μM, through the inhibition of the TrxR-Trx system and the disruption of the cell membrane. The Cys-X-X-Cys sequence was proposed to be the Au(I) binding site, but the specific binding site and cysteines involved in Gram-positive bacteria have not yet been fully elucidated. Similar to auranofin, silver compounds were also discovered as TrxR inhibitors against AMR. It was demonstrated for the first time that Ag(I) binds to the active sites of TrxR and the thioredoxin of *S. aureus* with dissociation constants of 1.4 ± 0.1 and 15.0 ± 5.0 μM, respectively.^46^ Binding with TrxR and thioredoxin disrupts the transfer of electrons from NADPH and inhibits the TrxR-Trx system in *S. aureus*. In addition to gold compounds, silver-based compounds and silver nanoparticles (AgNPs) were also considered to be TrxR inhibitors.^47^ For example, the *N*-heterocyclic carbene (NHC) silver acetate complex SBC3 (Figure 4h) was synthesized and reported as an antibacterial agent against *S. aureus*, including resistant *S. aureus* (MRSA) strains with an MIC value of 5.3–22 μM.^48^ It was further demonstrated that SBC3 exhibited antibacterial activity through inhibition of the bacterial thioredoxin reductase TrxR.^49^*E. coli* TrxR was reversibly inhibited by SBC3 in a dose-dependent manner, while the human TrxR mutant was not affected. Furthermore, it was reported that Ag(I) acts synergistically with ebselen, a selenazol antibacterial drug, against multi-drug-resistant Gram-negative bacterial infections with a Bliss score of 0.5 upon the cotreatment for 4 h.^50^ Ag(I) and ebselen have been found to exhibit direct inhibitory effects synergistically on *E. coli* TrxR and deplete GSH, leading to an accumulation of ROS, ultimately inducing cell death. Some metal-based anticancer drugs, e.g., Pt(II) and Ru(II) complexes, which exhibit inhibitory ability against TrxR of mammalian cells,^51,52^ have also been found to inhibit the growth of Gram-positive bacteria by an unclear mechanism. Although bacterial TrxR has a lower molecular weight compared to mammalian TrxR, it consists of similar Cys residues at the active site and still can be considered to be a possible target for Pt(II) and Ru(II) complexes to yield antibacterial abilities. Nevertheless, enhancing the biocompatibility and minimizing the side effects of metallo-antimicrobials is still a challenge since many essential enzymes are conserved between mammalian cells and pathogens. There is still a gap to fill for novel metal-based enzyme inhibitors prior to clinical application.

## Multiple Protein Inhibitors as Revealed by Metalloproteomics

**Figure 6:**
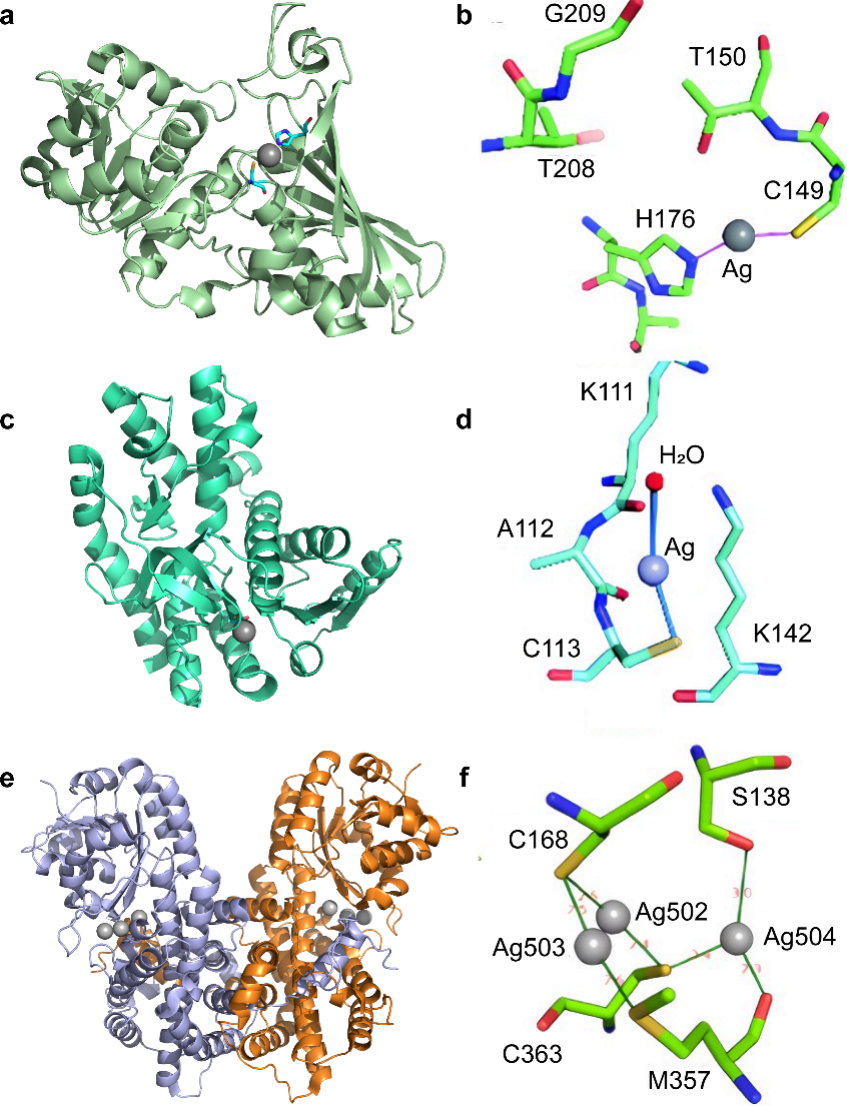
Crystallographic analysis of the binding mode of Ag(I). (a) Overall structure of Ag-bound GAPDH (Ag-GAPDH-1, PDB ID: 6io4). (b) Coordination geometry of Ag^+^ in the active site of Ag-bound GAPDH. (c) Overall structure of the Ag-bound MDH (Ag-MDH-1, PDBID: 5z3w) with Ag(I) shown as a sphere. (d) Ag(I) binds Cys113 and a water molecule. (e) Ribbon structure of Ag-bound 6PGDH (PDBID: 7cb6) with each monomer being highlighted in pale blue and brown, respectively. (f) Ag(I) ions bind critical residues at the active site of 6PGDH. The argentophilic interactions of adjacent Ag(I) were observed with Ag·g·g·Ag distances of ∼2.8 Å. Ag(I) ions are shown as spheres in (a), (c), and (e). The figures are partially generated by PyMol and partly adapted from ref (61) (available under a CC-BY 4.0 license; copyright 2018, the author(s)), ref (62) (available under a CC-BY 4.0 license; copyright 2021, the author(s)), ref (63) (reproduced with permission ref (63), copyright 2019, the Royal Society of Chemistry; permission conveyed through Copyright Clearance Center, Inc.), and ref (64) (reproduced with permission from ref (64); copyright 2020, the Royal Society of Chemistry; permission conveyed through Copyright Clearance Center, Inc.).

Given the multitargeted feature of metal-based drugs, it is highly desirable to track potential targets of a metallo-drug on a proteome-wide scale (e.g., cells and tissues). Metalloproteomics aims to systematically identify large sets of proteins associated with metals (metallo-proteins and metal-binding proteins) and their involvement in disease states and physiological processes.^53^ With the application of metallo-proteomics, the discovery of potential targets of metallo-antimicrobials has been accelerated.^54,55^ For example, bismuth-binding proteins in *Helicobacter pylori* were identified by integrative metallo-proteomics approaches combined with ICP-MS,^56−59^ allowing in total 63 Bi-binding proteins to be identified.^60^ By integration with proteomics, it was found that bismuth drugs disrupted multiple essential pathways in the pathogen, including ROS defense and pH buffering, by binding and functional perturbation of a number of key enzymes. Furthermore, by integration of liquid chromatography (LC) with GE-ICP-MS system (i.e., LC-GE-ICP-MS), silver(I) proteomes were tracked for the first time from *E. coli*(61) and *S. aureus*.^62^ In *E. coli*, Ag(I) was found to primarily target the oxidative branch of TCA cycle via functional disruption of the key enzymes, followed by adaptive glyoxylate cycle and subsequently abolishes bacterial oxidative defense ability vis inactivating key antioxidant enzymes. Binding of Ag(I) to key enzymes from TCA cycle including glyceraldehyde-3-phosphate dehydrogenase (GAPDH) (Figure 6a) and malate dehydrogenase (MDH) (Figure 6b) was further validated *in vitro*.^63,64^ The Ag(I) ions in the Ag-GAPDH-1 structure are located within a solvent-inaccessible site and coordinated by Cys149 and His176. And in the crystal structure of Ag-MDH-1, the Ag(I) ions binds to the Cys113 site (Figure 6c, 6d). Importantly, coadministration of silver(I) with metabolites in Krebs cycles such as citrate, significantly potentiates the antimicrobial efficacy of silver(I) and reduce the MIC of AgNO_3_ against *E. coil* from 1.8 to 0.6 μM.^61^ This study is an excellent showcase of how knowledge of the molecular mechanism of action of a drug can be harnessed to enhance the efficacy of the drug. While in *S. aureus*, silver(I) exhibits a different mechanism of action. Silver primarily targets glycolysis via functional disruption of multiple enzymes and generates ROS at the late stage, resulting in a metabolic divergence from glycolysis to the oxidative pentose phosphate pathway (oxPPP). However, such a metabolic divergence is ultimately futile owing to silver inhibition of key oxPPP enzymes e.g. Pgl and 6PGDH (Figure 6e).^62^ Five Ag(I) ions were identified to bind with 6PGDH with three of them binding to Cys168 and Cys363. Moreover, Ag^+^ and AgNPs were shown to be able to not only potentiate the efficacy of a broad range of antibiotics, resensitize MRSA to antibiotics, but also slow down the evolution of antibiotic resistance in *S. aureus*, highlighting a promising combination therapy of antibiotics with Ag(I) or other metal compounds to prevent occurrence of AMR and resensitizing the clinically used antibiotics.

For further discovery of metallo-antimicrobials, other metal ions with similar coordination features to silver(I) may also exhibit potentials to target GAPDH in glycolysis. For example, cathepsins, a family of lysosomal cysteine proteases responsible for intracellular proteolysis, are promising targets for cancer and antimicroorganism therapy. Cathepsin B and cathepsin K can be inhibited by many metal complexes including Au(I), Ru(II), Au(III), Pd(II) and Re(V), which have been comprehensively reviewed.^65^ Pt(II) complexes have also been found to inhibit cysteine proteases with IC_50_ values of 8–170 μM by binding to the active site.^66^ The knowledge of the mechanism of action of a drug may guide the identification of new targets for the design of inhibitors. A typical example is the discovery of a urease accessary protein, UreG as a novel target for the design of new type of urease inhibitor.^67^ Such a discovery is based exclusively on the understanding of mode of action of bismuth drugs toward inhibition of urease activity, which is indirect through disruption of urease maturation process owing to the binding to UreG. Therefore, we anticipate that with the application of metallo-proteomics, new druggable targets will be identified, which will render more antimicrobial agents to be designed and developed.

## Metalloagents as Membrane Perturbants

The cell membrane serves as a crucial barrier, regulating the transport of molecules, maintaining the cell’s structural integrity, and protecting against environmental pressure.^68^ The cell membrane of Gram-negative bacteria is composed of a peptidoglycan layer followed by a hydrophobic lipid layer containing lipopolysaccharides (LPS) in the out leaflet, which restricts the penetration of the hydrophobic antibiotics. In Gram-positive bacteria, a thicker peptide glycan layer containing teichoic acid is produced since the outer LPS barrier is absent, to protect the cell from the outer survival pressure and inhibit the penetration of many hydrophobic antibiotics.^69^ Consequently, binding and disrupting the integrity of bacterial membranes has been demonstrated as an effective strategy for developing new antibacterial therapies.^70^ Importantly, metallato agents serve as promising membrane perturbants due to the positively charged metal ions, which have high affinity for negatively charged molecules in the cell membrane. Upon binding to cell membrane proteins, metallo-antimicrobials are able to disrupt the membrane integrity, causing damage and leakage that ultimately leads to cell death.

**Figure 7:**
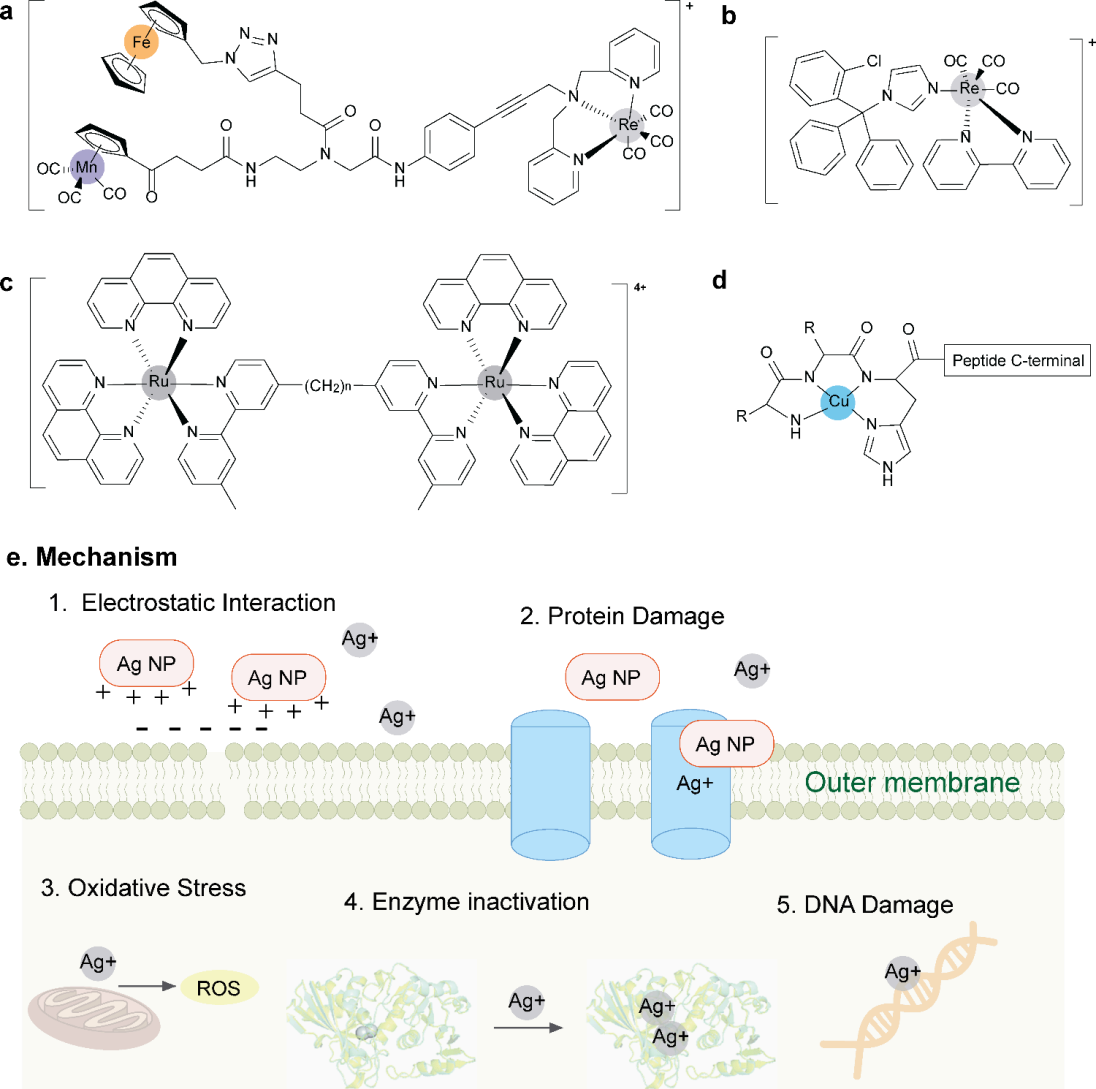
Chemical structures of (a) **Re1**, (b) Ru(II) complex, (c) **Re2**, and (d) **Cu-ATCUM** complex. (e) Scheme illustrating the proposed mechanism of nanoparticles as membrane perturbants. AgNPs disrupt the cell membrane and generate ROS and the released Ag^+^ damage protein as well as cause DNA damage.

One of the most effective metal-based membrane perturbates is metallo-nanoparticles (NPs), which not only have the ability to bind and disrupt bacterial membrane, but also bind to intracellular components, including DNA, ribosomes and enzymes, causing the production of ROS (Figure 7).^71^ The antibacterial mechanisms of metallo-NPs,^72^ metal oxide NPs,^73^ and other NPs have comprehensively been reviewed.^74^ Herein, we mainly focus on the metallo-based NPs membrane perturbants. Metallo-NPs can accumulate and adhere to the bacterial cell wall by electrostatic attractions, van der Waals, and hydrogen bonding interactions due to their unique chemical properties.^75^ For example, AgNPs exhibited promising antibacterial activity and synergistic effect with vancomycin with MIC ranging from 8 to 16 μg/mL by causing membrane damage and leakage.^76,77^ The accumulation of AgNPs at the cell surface causes depolarization of the cell wall, alters the zeta potential, and increases the membrane permeability of *S. aureus*(78) and *E. coli*.^79^ Metallo-NPs also function by releasing free metal ions as active agent. For example, AgNPs can increase membrane permeability by disrupting disulfide bond formation and misfolded protein secretion.^80^ The released Ag(I) exhibits high binding affinity toward thiolate groups, thereby disrupting the formation of disulfide bonds that are important to maintain membrane integrity. Transmission electron microscopy (TEM) confirmed significant morphological changes in the cell envelope, while the uptake of the membrane-impermeant dye propidium iodide was increased.^79^ In addition, Ag(I) can induce oxidative stress and interfere with bacterial metabolic processes, accountable for the antimicrobial activity. The ability of AgNPs to release free Ag(I) ions gives them relatively better antibacterial abilities with MIC values of 0.01–0.2 pM against *S. aureus* 29213 and *E. coli* 25922 comparing with its metal oxide NPs (MONPs).^81,82^ Some MONPs also showed antibacterial activity as membrane perturbants by interacting and penetrating through the cell membrane.^83^ Interestingly, AgNPs exhibited synergistic effects with some antibiotics such as kanamycin^84^ and tetracycline^85^ against *S. aureus* with FIC index values less than 0.5. Metallo-NPs in combination with clinically used antibiotics could enhance the antibiotic activity, reducing the development of resistance and providing a broader spectrum against a range of pathogens. However, one inevitable problem to face before translating metallo-NPs to clinical usage is potential toxicity in humans. Until now most of the metallo-NPs that exhibited great antibacterial activity have relatively high cell toxicity. Since both the bacterial cell and mammalian cell are positively charged, negatively charged NPs accumulated and caused cell leakage without any selectivity. The development of metallo-NPs selectively targeting bacteria or pathogens is one of the potential strategies to reduce their cell toxicity. For example, peptidoglycan is a unique macromolecule that is not found in eukaryotic cells, making it a possible target for treating Gram-positive bacterial infections.

Other metal compounds, including organometallic compounds, also exhibit antibacterial abilities as a membrane perturbant. **Re1**, a heterotriorganometallic PNA monomer derivative with Fe, Mn, and Re in its structure (Figure 7a), could induce membrane depolarization and reduced cytosolic ATP levels, indicative of its specific interaction with the cytoplasmic membrane. The Re(I) moiety is the key fragments for **Re1** to exhibit its antibacterial activity, while the ferrocene and manganese fragments contributing to the oxidative stress are replaceable.^86^ Similarly, the complex **Re2** (Figure 7b) could inhibit Gram-positive bacteria, including MRSA, with MIC values of 0.3–2.6 μM, by multiple mechanisms including membrane insertion, membrane disorganization, inhibition of peptidoglycan synthesis, release of CO, and disruption of the membrane potential.^87^ Ru(II) complexes with long linkers (Figure 7c) are capable of interacting with bacterial membranes, as confirmed by solid-state NMR and molecular dynamics simulations.^88^ These Ru(II) complexes can insert into the negatively charged model bacterial membrane, resulting in an increased disorder of lipid acyl chains and membrane-thinning, which is important for membrane permeability and the uptake of other antibiotics. In addition to metal-based NPs and metal complexes, metallo-antimicrobial peptides (metallo-AMP) are recently investigated to boost the antibacterial activity of AMPs. AMPs are naturally occurring peptides serving as essential innate host defense effector molecules.^89^ A subclass of AMP, containing an amino terminal Cu(II) and Ni(II) (ATCUN) motif, is able to bind both Cu(II) and Ni(II) ions with high affinity and deliver the metal ions into the targeting site, resulting in a synergistic enhancement.^90^ The Cu-ATCUN (Figure 7d) complex has been shown to have MICs as low as 0.3 μM against *Enterococcus faecium*(91) and to exhibit catalytic properties that produce reactive oxygen species (ROS) via the Cu(II)/Cu(III) redox chain at the membrane site. This process results in more damage to the membrane compared with its parent AMP, thereby contributing to a synergistic effect in antimicrobial activity against *E. coli*. Till now, Cu(II)-AMP complex appears to be the only one, that has been proposed as a potential arsenal against AMR, however little is yet known about the ATCUN motif, which provides a novel therapeutic opportunity to conjugate metal ions with AMPs by inserting a metal-binding motif to increase the efficiency of the membrane disruption.

## Metalloantimicrobial Agents Targeting the Uptake/Efflux System

### Efflux Pump Inhibitors/Regulators

Efflux pumps are possibly the fastest and most effective resistance mechanism in bacteria that can rapidly export antibiotics and other toxic compounds actively from the bacterial cell, reducing their intracellular concentration and allowing bacteria to survive.^92^ Bacterial efflux pumps are classified into six families: the ATP-binding cassette (ABS) family, the major facilitator superfamily (MFS), the multidrug and toxin extrusion (MATE) family, the small multi-drug-resistance (SMR) family, the resistance-nodulation-cell division (RND) superfamily and the proteobacterial antimicrobial compound efflux (PACE) family.^93^ The overexpression or upregulation of efflux pumps is a common mechanism by which bacteria develop resistance to multiple antibiotics, leading to multidrug resistance (MDR). Consequently, inhibiting efflux pumps or inhibiting the overexpression of efflux pump genes has emerged as a promising strategy to combat AMR.

Hexaamminecobalt (HC) is reported as one of the efflux pump inhibitors that directly bind with proteins and alter the structure of the tripartite efflux pump TolC in *E. coli*.^94^ HC binds to TolC tightly with a *K*_d_ value of 0.74 μM. Although the binding blocked the activity of TolC, it does not exhibit antimicrobial activity or any synergistic effect with antibiotics. Many metallo-NPs have been shown as efflux pump gene regulators. For example, functionalized ZnO@Glu-TSC as an efflux pump inhibitor by conjugation of thiosalicylic acid (TSC) with ZnONPs to treat multidrug resistant *S. aureus* infections.^95^ ZnO@Glu-TSC demonstrated antibacterial activity against ciprofloxacin-resistant *S. aureus*, with MIC values ranging from 8 to 512 μg/mL. The combination of ciprofloxacin with ZnO@Glu-TSC showed a 32-fold enhanced activity compared to ciprofloxacin alone. The expression of efflux pump genes *norA*, *norB*, *norC*, and *tet38*, which are important for multidrug resistance (MDR), were significantly reduced upon treatment with ZnO@Glu-TSC. Similarly, ZnO-NPs inhibit the NorA efflux pump in *S. aureus* with 27% increase in the inhibition zone comparing with ciprofloxacin against *S. aureus*.^96^ ZnO-NPs was also shown to inhibit the bacterial growth (MIC of 31 μg/mL) against *A. baumannii*, and regulated the *AdeB* and *AdeRS* gene expression, which are related to the efflux pump function in *A. baumannii*.^97^ In addition, cobalt-doped ZnO-NPs coated with thiolate chitosan exhibited antimicrobial activity against *S. aureus* with a MIC value of 10 μg/mL, attributable to synergistic inhibition of the efflux pump of *S. aureus* with thiolate chitosan.^98^ Besides ZnO-NPs, AgNPs have also been shown to downregulate the expression of efflux pump genes, including *AdeA*, *AdeC*, *AdeS*, *AdeR*, *AdeI*, *AdeJ*, and *AdeK*, against multidrug resistant *A. baumannii* strains with MIC values ranging from 25 to 200 μg/mL.^99^ AgNPs were also demonstrated as an effective efflux pump inhibitor against *Burkholderia pseudomallei* with MIC value of 32 μg/mL.^100^ CuNPs can also function as efflux pump inhibitors through inhibition of NorA in *S. aurues*.^101^ There is a 4-fold enhancement in the MIC of ciprofloxacin (from 20 to 5 μM) against multi-drug-resistant *S. aureus* when combined with CuNPs. Padwal et al. synthesized Poly(acrylic acid)(PAA)-coated iron oxide (magnetite) NPs (PAA-MNPs) were synthesized and demonstrated to be an efflux pump inhibitors against *Mycobacterium smegmatis* for treating tuberculosis (TB).^102^ The combination of PAA-MNPs and rifampicin exhibited a 4-fold improvement compared to rifampicin alone (from around 40 to 4 μM). This synergistic effect is attributed to the inhibition of the efflux pump by PAA-MNPs, resulting in the 3-fold increase in the accumulation of rifampicin in the cells. Metallo-antimicrobials have shown potential in inhibiting efflux pumps, thus sensitizing bacteria to antibiotics through combination therapy.^96^ By targeting and blocking the activity of efflux pumps responsible for antibiotic extrusion, the efficacy of antibiotics can be restored, offering a promising approach to overcome AMR. Further research is necessary to optimize the design and effectiveness of metallo-based efflux pump inhibitors to evaluate their potential for clinical applications.

### Metallosiderophore Complexes

**Figure 8:**
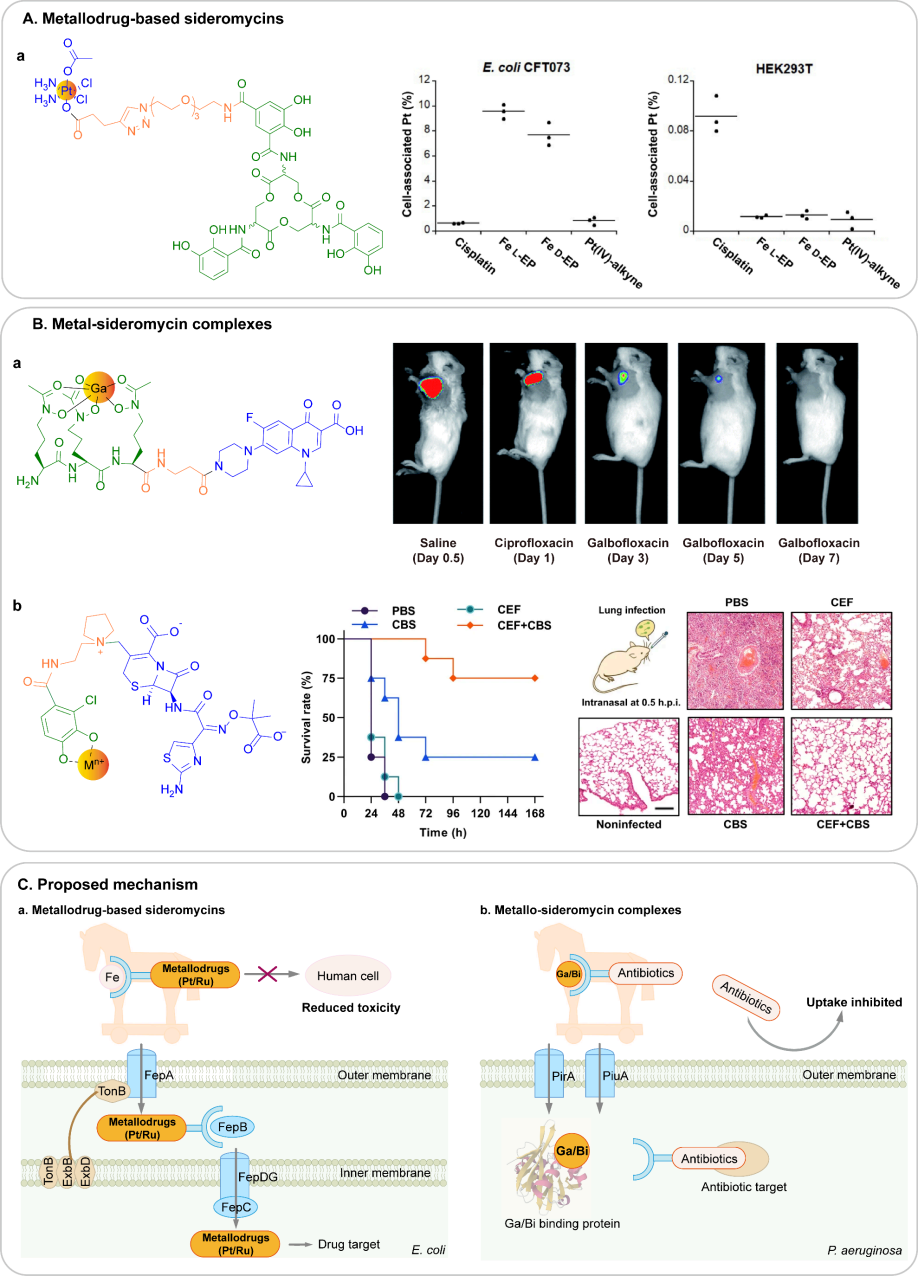
Trojan horse strategy for delivery of antimicrobial agents. (A) Metallo-drug-based sideromycins. (a) Chemical structures of enterobactin-Pt(IV) conjugates and its selectively cellular uptake in bacterial cell *E. coli* CFT073 and mammalian cells HEK293T. (B) Metallosideromycin complexes. (a) Chemical structure of the Ga(III) complex of ciprofloxacin-functionalized desferrichrome and the enhanced efficacy against *S. aureus in vivo*. (b) Proposed chemical structure of a metallo-sideromycin using cefiderocol (CEF, S-649266) as an example. CBS enhances the antibacterial activity of CEF against PAO1 *in vivo*. (C) Proposed mechanisms of (a) metallo-drug-based sideromycins and (b) metallo-sideromycin. The figures are adapted from ref (111) (copyright 2022 American Chemical Society), ref (113) (reproduced with permission from ref (113); copyright 2021, the Royal Society of Chemistry; permission conveyed through Copyright Clearance Center, Inc.), and ref (115) (available under a CC-BY 4.0 license; copyright 2023, the author(s)).

Iron is an essential nutrient for microbials and serves as a cofactor of many enzymes involved in key biological processes. The acquisition of iron (as Fe(III)) is a critical factor for bacterial growth due to its low solubility and competition from the host environment.^103^ Bacteria produce siderophores, low-molecular-mass (400–2000 Da) Fe(III) chelators, to scavenge iron from the host environment and uptake enough iron to prevent iron starvation and sustain themselves.^104^ Siderophores exhibit a high affinity toward Fe(III) and form strong complexes with Fe(III) by chelation with key moieties including catecholates, hydroxamates and carboxylates, as well as some mixed moieties containing more than one of the mentioned binding moieties.^105^ The “Trojan horse” strategy, which utilizes bacterial siderophore transport systems as a cargo to deliver antibiotics across the outer membrane barrier of Gram-negative bacteria and peptidoglycan layer of Gram-positive bacteria, has been proposed as a promising approach for the development of novel antibiotics and the restoration of the activity of clinically used antibiotics.^106−109^ These natural and synthetic siderophore-drug conjugates, also called as sideromycins, exhibit improved antimicrobial activities by hundred-folds compared to their parent antibiotics.^108^ Metal-based siderophore-drug conjugates are being actively investigated due to the selective uptake of siderophores by bacterial cells, which can help to reduce drug toxicity in human cells. One of the strategies is metallodrug-based sideromycins, which use siderophores to link to metal-based drugs as antibacterial agents, thus reducing the cell toxicity in mammalian cells, importantly enhancing the antibacterial abilities against Gram-negative bacteria (Figure 8Ca). A ruthenium-based Trojan horse drug as a novel antibacterial agent was synthesized.^110^ Four Ru(III)-siderophore conjugates exhibit antibacterial activities against *E. coli*, *K. pneumonia*, *P. aeruginosa*, and *A. baumannii*, with very low toxicity to human ovarian carcinoma and human embryonic kidney cells (IC_50_ > 200 μM). Cisplatin is one of the most well-known anticancer drugs, exhibiting promising anticancer abilities but also high toxicity toward human cells, which is an obstacle for clinical application in the treatment of both cancer and bacterial infections. A heavy metal Trojan horse was designed using enterobactin to deliver Pt(IV) drugs directly into *E. coli* cells to fight against bacterial infections.^111^ The enterobactin-Pt(IV) conjugate (Figure 8Aa) exhibited antibacterial activity against *E. coli* K12, but very low uptake (∼0.01%) by human embryonic kidney cells compared to cisplatin (∼0.01%), suggesting a strategy for drug repurposing by using siderophore to enhance the drug uptake into bacteria and to avoid the cell toxicity of heavy metal drugs to the hosts. Since the hexadentate siderophore showed very tight binding affinity to Fe(III), other metal ions with similar electron structure to iron were also taken into consideration to coordinate tightly to the siderophore, in other words, to mimic the iron-siderophore complex and deliver both metal and antibiotics as metallo-sideromycin complexes into bacterial cells (Figure 8Cb). A Ga(III) complex of ciprofloxacin-functionalized desferrichrome (namely Gabofloxacin) was reported (Figure 8Ba) as a diagnostic drug against *P. aeruginosa*, *S. aureus*, and *K. pneumoniae* strains with MICs ranging from 0.94 to 12.5 μM.^112^ It exhibited a superior potency against *S. aureus* with a MIC value of 0.093 μM both *in vitro* and *in vivo*.^113^ Similarly, a Salmochelin S4-inspired ciprofloxacin conjugate showed good activity against *E. coli* K12 and Nissle 1917 with MICs of 75 μM and 100 μM.^114^ When ^67^Ga(III) was conjugated with salmochelin-ciprofloxacin sideromycin, antibacterial activity was increased in both iron-sufficient and iron deficient conditions. In addition, we recently reported that Bi(III) showed strong synergistic effect (FIC of 0.125) with cefiderocol, an FDA approved sideromycin, against *P. aeruginosa*.^115^ Bicefiderocol complex was formed in a 1:1 ratio (Figure 8Bb) and exhibited enhanced antibacterial activity with MIC of 1 μM against cefiderocol-resistant strains compared with cefiderocol itself with MIC of 16 μM. The MIC of cefiderocol (CEF) could be reduced by 64-fold (from 4 to 0.0625 μM) with the combination of CBS, and the development of high-level bacterial resistance to CEF was suppressed. Moreover, the synergistic effect is proved in a murine lung infection model, implicating the high clinical translational potentials. In summary, metallo-drug-siderophore conjugates and metallo-sideromycin complexes showed enhanced antimicrobial activities to overcome antibiotic resistance, and at the same time reduce the cell toxicity of heavy metal drugs. Metallo-sideromycins have the potential to not only enhance the efficacy of sideromycins but also prolong their effective life-span as antibiotics. Therefore, it is worthwhile to further investigate other sideromycins and metals to thoroughly explore the potentials of metallo-sideromycins in fighting against AMR.

## Metallo-antimicrobial Agents as Persister Inhibitors

### Biofilm Inhibitors

One of the biggest challenges for the treatment of bacterial infections is the long-term persistence infections caused by persister cells, which can survive in the hosts for a long period of time with reduced metabolism and restart growth after antibiotic treatment.^116^ The persistence infections were first proposed in 1994 by Joseph who observed that *Staphylococcus* survive under the treatment of penicillin.^117^ Different from AMR, persistence is a reversible state that does not involve specific genetic mutations or protein modification at the cellular level. Persistent bacterial cells exhibit a phenotypic switch to a dormant and nonreplicating state, which makes them less susceptible to antibiotics.^118^ Persistence forms during the biofilm growth, which provides mechanical shelters for resident persister bacterial cells to protect the cells from environmental stress such as desiccation and antibiotic stress.^119^ These shelters are composed of lipids, polysaccharides, proteins, extracellular DNA (eDNA), and chemical signaling molecules with bacterial cells.^120^ Consequently, inhibiting biofilm formation is one of the effective strategies to combat persistence infections. There are four main strategies to combat biofilm-related AMR: (a) inhibit the initial microbial adhesion by coating antibiofilm materials; (b) interfere with the QS (quorum sensing) system or related signaling molecules; (c) disrupt or inhibit the extracellular polymeric substances or other biofilm composition production; and (d) inhibit the formation of persister cells and resensitize the persister cells to antibiotics.

**Figure 9:**
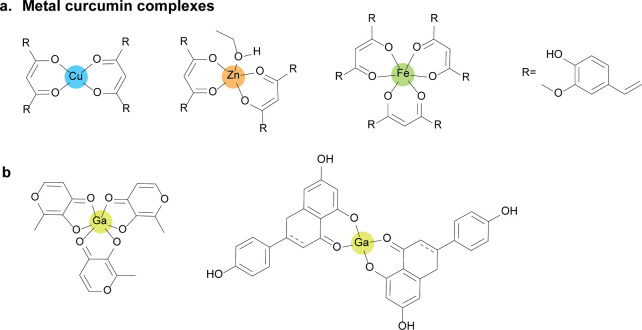
Chemical structures of quorum sensing inhibitors against *P. aeruginosa* infection. (a) Metal curcumin complexes and (b) Ga(III) complex with maltolate and flavonoid.

Metallo-antimicrobials hold great promise to be developed as antibiofilm compounds due to their advantages in entering the EPS matrix, which contains several weakly acidic groups to exhibit coordination properties and cation sorption with metal ions.^121^ Olar et al. recently summarized that transition-metal complexes including Mn(II), Ni(II), Co(II), Cu(II), and Zn(II) coordinating with multidentate ligands show antibiofilm activities.^122^ Metal-N-heterocyclic carbene complexes of Ag(I), Au(I), and Cu(I) (Figure 9a) were also shown to be able to inhibit biofilm formation against both Gram-negative bacteria *P. aeruginosa* and *E. coli* and Gram-positive bacteria *Listeria monocytogenes*, *S. aureus*, and *S. epidermis*.^123^ Ga(III) compounds such as gallium citrate, which has been investigated for years as antimicrobial agents, can remove the biofilm formed by *P. aeruginosa* at relatively low concentrations.^124^ Furthermore, in a preliminary phase I clinical trial, gallium was shown to enhance lung function in patients with cystic fibrosis (CF) and chronic *P. aeruginosa* lung infection.^181^ Among metal complexes with different synthetic ligands, antibiotic complexation with metal ions is one of the effective methods to restore or enhance antibiotic abilities against biofilms. The Zn-fluconazole complex showed antibiofilm abilities against *C. albicans* and *P. aeruginosa* strains, which was able to inhibit the biofilm formation by 20% in comparison to the untreated control.^125^ A Ga-flavonoid complex (Figure 9b) was reported to eliminate biofilm formation in *P. aeruginosa* though reducing the secretion of bacterial virulence factors, and the presence of 0.08 μM Ga(NO_3_)_3_ reduced biofilm formation by approximately 50% compared to the untreated control.^126^ It was also reported that direct combination therapy with metallo-antimicrobials such as Bi(III) and small-molecule antibiotics without complexation works well against biofilm formation. Bi(III) compounds (e.g., CBS) showed the ability to inhibit biofilm formation of *P. gingivalis* and disrupt the mature biofilm combined with metronidazole, thus eliminating the existence of persister cells in a biofilm.^127,128^ The combination of Bi(III) thiols (BTs) with ciprofloxacin showed a synergistic inhibitory effect against *P. aeruginosa* biofilm formation at a concentration of 12.5 μM. Pravibismane (MBN-101, bismuth ethanedithiol, BisEDT; topical) is being developed in a phase-II trial as a broad spectrum antibacterial with antibiofilm activity against diabetic foot infections.^182^ Ga(III) showed a synergistic antibiofilm effect with salicylidene acylhydrazide and toxin production by *P. aeruginosa* strains.^129^ Ga(III) chelated the hydrazone, enhanced the antibiofilm effect, and suppressed the type III secretion system in *P. aeruginosa*. Metal complexes as antibiofilm agents have several advantages including the multitargeted modes of action and the potential for imaging and diagnostics compared with small molecules. However, it remains a big challenge to unveil the mechanism of action of most metal complex-based biofilm inhibitors, which is an essential prerequisite for the clinical application of metallo-antimicrobials.

Metal nanoparticles and nanotechnologies also gained increasing attention as potential biofilm inhibitors due to their high surface area to volume area, allowing them to penetrate the EPS matrix and accumulate into biofilms. Ag and Au nanoparticles (AgNPs and AuNPs) are the most studied metal nanoparticles as antibacterial agents. AgNPs can inhibit biofilm formation and eliminate the mature biofilms of *A. baumannii*, *E. coli*, and *S. aureus*.^130^ The disintegration of AgNPs releases Ag(I) ions, which subsequently interact with the bacterial cell membrane, leading to depolarization of the cell wall and cell death. In addition, the Ag(I) ions can disrupt bacterial growth as a multitargeted antimicrobial agent as mentioned before, such as inhibiting the activity of metal-binding enzymes and producing the ROS.^61,62,80^ AgNPs are able to inhibit the biosynthesis of rhamnolipids by disrupting the quorum sensing system in *P. aeruginosa*(131) as well as decrease the transcription of biofilm-related genes *bap*, *OmpA*, and *csuA*/*B* in multi-drug-resistant *A. baumannii*.^132^ Similar to AgNPs, AuNPs exhibit antibiofilm ability by damaging the bacterial cell membrane and inhibiting the production of EPS.^133^ Moreover, AuNPs show photothermal abilities under near-infrared (NIR) light to cause damage by generating localized heat.^134^ Recently, bismuth nanoparticles, BiNPs, were also shown to exhibit antimicrobial activity against the emergent multi-drug-resistant yeast *Candida auris* under biofilm growing conditions^135^ as well as *S. gordonii* strains.^136^ Metal oxide NPs such as TiO_2_, ZnO, and CuO nanoparticles have shown promising antimicrobial properties against biofilm formation.^137,138^ ZnO@NP and ZnS@NPs inhibit biofilm formation and eliminate the mature biofilm by generating ROS to induce oxidative stress^139^ and interfere with the gene expression of the toxin–antitoxin system in *P. aeruginosa*.^140^ Furthermore, the composite nanoparticles were also considered to exhibit better antibiofilm abilities and bioavailabilities. A novel approach based on the multinuclear metal complex DNase-mimetic artificial enzyme (DMAE) was developed. It was prepared by passivating AuNPs with multiple Ce(IV) complexes on the surface of colloidal magnetic Fe_3_O_4_/SiO_2_ core/shell particles. This DMAE system inhibited biofilm formation by disrupting the integrity of EPS.^141^ Bismuth-organic frameworks showed prolonged inhibition against biofilm formation compared to colloidal bismuth citrate (CBS) owing to the sustained release of Bi(III) ions, indicative of the potential of MOFs (metal organic frameworks) against AMR.^142^ Regarding direct application in treating infections, metal-based nanotechnologies can also be used as coating materials to prevent initial microbial adhesion in medical and industrial locations.^143^ We believe that metal-based nanoparticles are unique potential candidates to be developed as biofilm inhibitors if cell toxicity and stability issues are well characterized.

### Quorum Sensing (QS) Inhibitors

**Figure 10:**
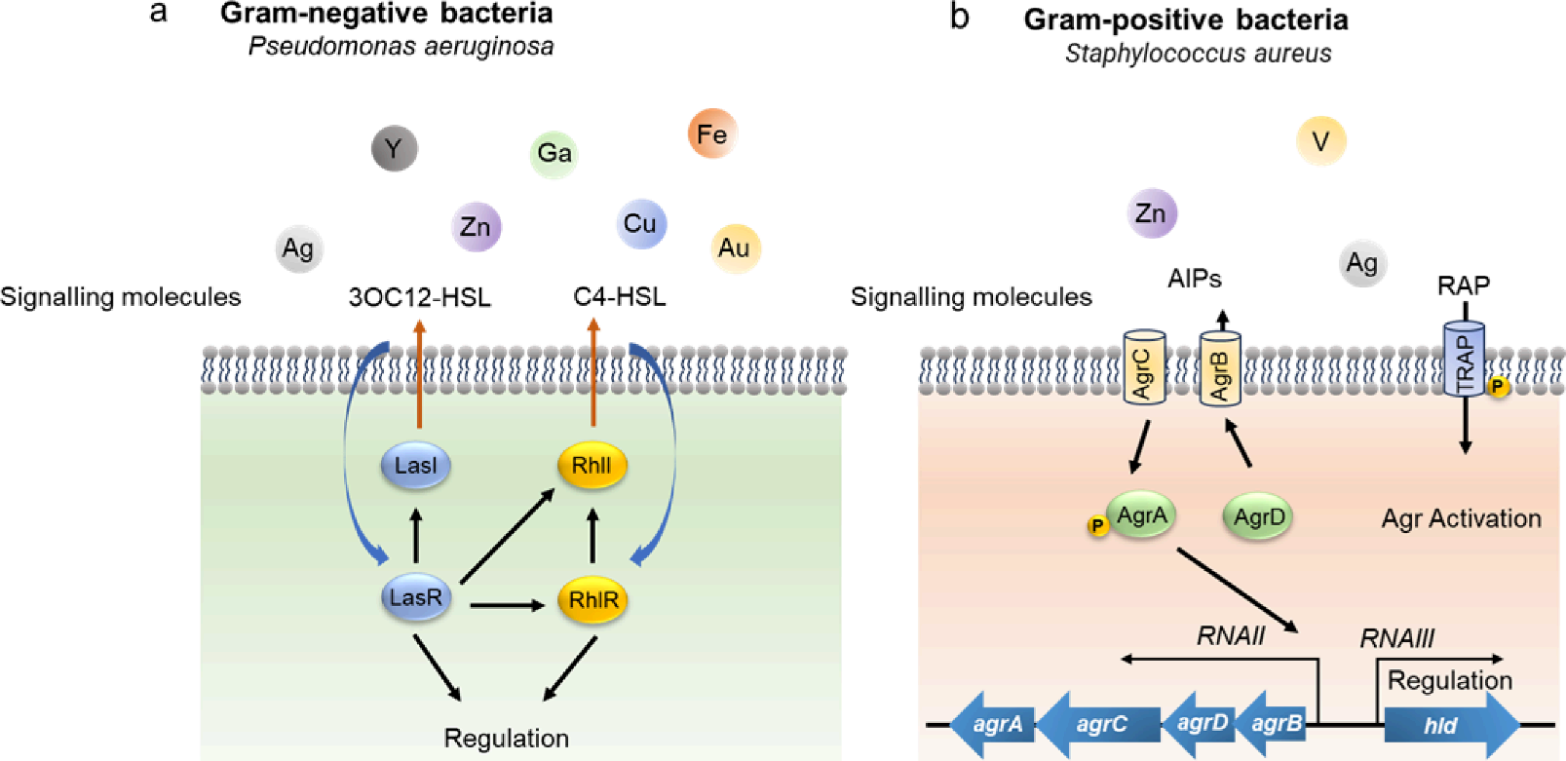
Metal-based agents as bacteria quorum sensing (QS) system inhibitors. (a) The reported Ag(I), Cu(II), Au(III), etc. target Lasl-LasR and RhlI-RhlR systems in Gram-negative bacteria to use antibacterial activity (i.e., *Pseudomonas aeruginosa*). (b) Zn, Ag, and V inhibit agar and RAP-TRAP systems in Gram-positive bacteria (i.e., *Staphylococcus aureus*).

The quorum sensing (QS) system is the communication process in bacteria that regulates adaptive activities to suit the environment better.^128,144^ Bacteria can express pathogenicity and develop persistence through QS-regulated virulence factors while the targeting and interfering QS system may control the serious pathogenicity and persistent infections.^145^ Importantly, the QS system plays a crucial role in biofilm formation. Bacteria release specific quorum sensing signaling molecules during biofilm formation to regulate the related gene expression such as EPS production.^146^ Consequently, inhibiting the QS system is a novel and important approach to developing antibacterial drugs. The metal-based strategy is receiving growing attention toward combating multi-drug-resistant pathogen virulence by inhibiting the QS system in both Gram-negative and Gram-positive bacteria, and many metallo-quorum sensing inhibitors (QSIs) have been reported as persistence breakers (Figure 10). Regarding Gram-negative quorum-sensing bacteria, the quorum sensing systems of *P. aeruginosa* use two types of autoinducing chemical signaling molecules, N-acylhomoserine lactone (AHL) and 4-quinolones (4Qs).^147^ Y(III), Ag(I), Bi(III), Cu(II), Zn(II), Fe(III), and Au(III) complexes and nanoparticles were identified to target these two signaling molecules and inhibit QS activity in Gram-negative bacteria. AgNPs, which have been used widely for treating bacterial infections, show inhibitory abilities to *rhIR* in *P. aeruginosa*.^148^ It was demonstrated that yttrium oxide core/shell nanospheres mitigated bacterial quorum sensing, virulence functions, biofilm formation, and the expression of transcription regulatory quorum sensing gene (*rhIR*) in drug-resistant *P. aeruginosa* isolates.^149^ Bismuth porphyrin complexes was found to be effective inhibitors of the *P. aeruginosa* QS system through the suppression of 3-oxo-C12-HSL production.^150^ PtNPs and PdNPs can inhibit the QS system through interaction with LasR.^151^ Metal-curcumin complexes were observed to exhibit an antiquorum sensing activity of *P. aeruginosa* PAO1. In particular, the Cu(II)-curcumin complex exhibited the best inhibitory effect on swarming and twitching motilities, biofilm formation, and alginate and pyocyanin production. It also has sensitivity to H_2_O_2_ and reduction in the expression levels of *lasI* and *lasR* genes.^152^ A Ga(III) complex with maltol (GaM) was also reported to be able to downregulate the QS system in *P. aeruginosa*.^153^ Recently, we have demonstrated that a Ga(III)-flavonoid complex exhibits potent antibacterial activity by interfering with the QS system and iron metabolism. Our transcriptomic analysis revealed that only the *lasR* gene in the QS system was significantly upregulated.^126^

Gram-positive bacteria such as *S. aureus* have different types of QS systems with autoinducing peptides (APIs) serving as signaling molecules compared with Gram-negative bacteria. To regulate virulence, *S. aureus* employs two distinct QS systems (i.e., the AGR system and the RAP/TRAP system).^154^ There are also many effective metallo inhibitors targeting the Gram-positive bacterial QS system. A novel antimicrobial coating consisting of Ag and Ru efficiently inhibited MRSA growth through downregulating the biofilm-related gene and QS system gene.^145^ A nalidixic acid-vanadium complex (V-NA) loaded into chitosan hybrid nanoparticles was synthesized as a quorum sensing inhibitor against both Gram-positive bacteria and Gram-negative bacteria.^155^ Complexes of Zn(II) and Ag(I) with 2-trifluoroacet-onylbenzoxazole ligands inhibited the QS system of *S. aureus*.^156^ However, it remains unclear how metallo-antimicrobials interfere with QS. It is important to point out that some studies lack proper controls and utilize conditional growth media to regulate the levels of essential elements for biofilm growth. While these foundational studies have provided us with innovative ideas and confidence in the potential of metallo-antimicrobials as QS inhibitors, further research should prioritize a more thorough investigation to identify promising avenues for exploration.

## Metallo-antimicrobial Agents as Oxidative Stress Inducers

### Photosensitizers

**Figure 11:**
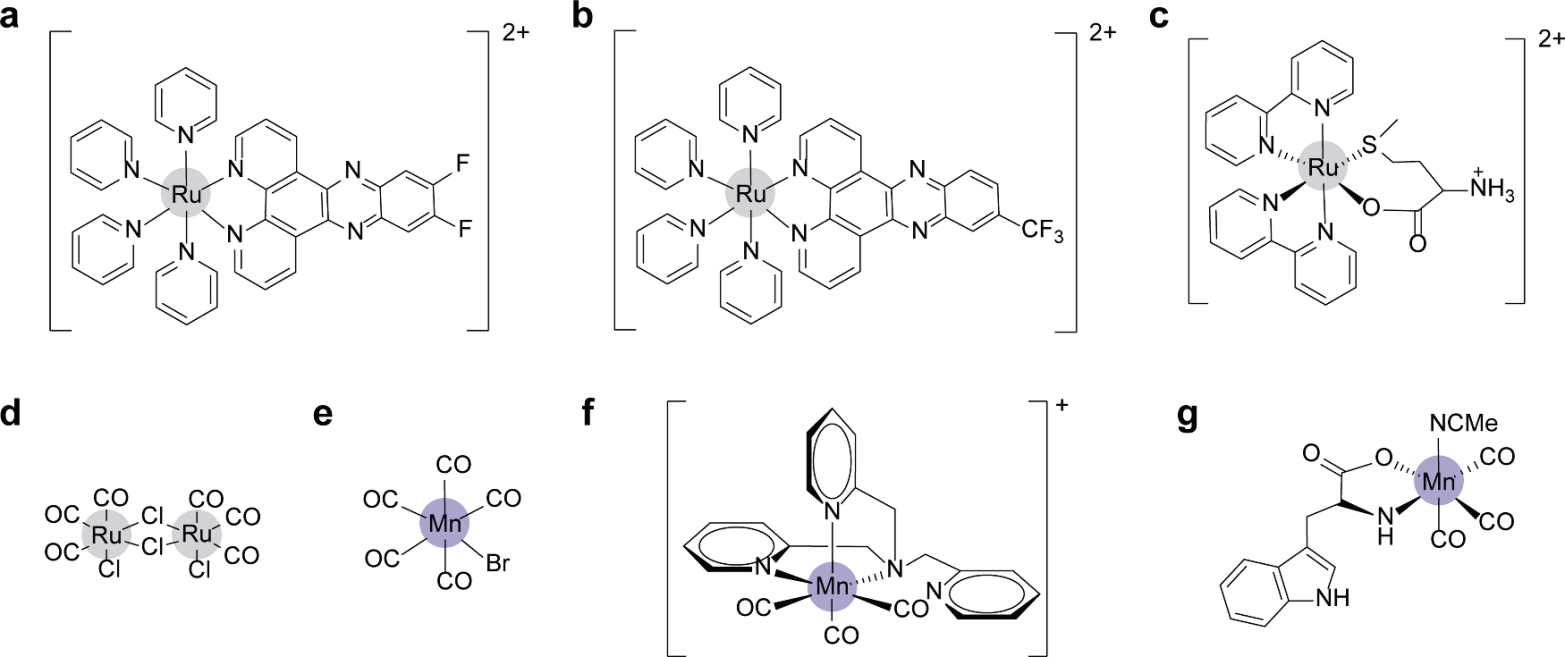
Chemical structures of (a–c) photoactivated Ru(II) complexes, (d) Ru(II) CORMs, and (e–g) Mn(I) CORMs.

Reactive oxygen species (ROS) damage is one of the most common mechanisms of action of metallo-drugs in inhibiting bacterial growth. ROS including superoxide anions, hydrogen peroxide, and hydroxyl radicals are highly reactive molecules that can damage proteins, lipids, and DNA. However, oxidative stress also poses a significant threat to eukaryotic cells, presenting a challenge for metallo-antimicrobials to selectively induce cellular ROS in pathogens. This dilemma underscores the potential of therapeutic strategies that target microbial oxidative stress responses without a harmful influence on host cells. Antimicrobial photodynamic therapy is a rapidly growing area to fight against AMR.^157^ Based on the surface structural and specific life processes of bacteria (e.g., membrane charge, cell wall compositions, membrane proteins), several strategies have been reported to design photosensitizers selectively targeting bacteria.^181^ Combined with metallo-antimicrobials, metal-based photosensitizers such as metal porphyrin complexes showed improved photophysical and biological properties compared to the ligands. Ru(II),^158^ Ir(III),^159^ Pt(II),^160^ Cu(I),^161^ and Ga(III)^180^ complexes were reported as effective metal antibacterial photosensitizers. These metal complexes exhibited antibacterial abilities through not only light-triggered ROS production but also the light-triggered release of metal ions from the ligand. For example, two photoactivated Ru(II) complexes (Figure 11a,b) were demonstrated to exhibit potent antibacterial activity against MRSA, vancomycin-resistant *Enterococcus* (VRE), and *E. coli* with MIC values ranging from 2 to 8 μM upon irradiation.^162^ The *in situ*-released Ru(II) aqua complexes upon the phototriggered ligand dissociation can covalently bind to DNA, synergistically inhibiting bacterial growth by singlet oxygen production through a photodynamic mechanism. Specifically, a Ru(II) methionine complex (Figure 11c) binds to DNA after blue light irradiation, resulting in efficient DNA cleavage.^163^ This complex was exposed to blue and green light photolysis (at 453 and 505 nm, respectively) in an aqueous solution, leading to the release of methionine and the formation of the cis-[Ru(bpy)_2_(H_2_O)_2_]_2_^+^ ion. This photoproduct was found to interact with DNA thereafter, leading to DNA photocleavage.

Metal photosensitizers address the limitation of photodynamic therapy (PDT) against AMR under hypoxic conditions and somehow reduce the cytotoxicity of metal ions to eukaryotic cells by light-triggered *in situ* metal release. In addition, metal NPs also demonstrated potential for disinfecting surfaces and destroying biofilms through the photodynamic mechanism.^164^ Although the PDT strategy may be limited to surface infections due to the light penetration problem, it is inspiring that the generation of ROS and the release of metal can be controlled precisely through light irradiation, thereby helping to prevent off-target toxicity. These macrocycles and nanoparticles may also serve as platforms for introducing metal ions into targeted bacterial cells by a fine-tuned ligand design.

### CO/NO Releasers

Carbon monoxide-releasing molecules (CORMs) have been studied for their potential antimicrobial properties by enhancing the activity of CO and triggering oxidative stress.^165^ CORMs are capable of releasing controlled amounts of carbon monoxide (CO) when triggered by specific conditions, such as light activation, enzymatic action, or changes in pH. Metal-based CORMs were demonstrated to possess biological activity on multi-drug-resistant Gram-negative bacteria. At appropriate therapeutic concentrations, the released carbon monoxide (CO) can exhibit selective toxicity against bacteria while being harmless to normal tissue.^166^ Ru(II) and Mn(I) CORMs (Figure 11d,e) were first reported as antimicrobial agents against *S. aureus* and *E. coli* by releasing CO gas.^166^ Furthermore, a series of Mn(I) tricarbonyl complexes (Figure 11f) could achieve controllable CO release by photoactivation with MIC values in the range of 100 μM against *P. aeruginosa* strains.^167^ A visible-light-induced Mn(I) CORM (Figure 11g) was found to exhibit a potent antibacterial effect against *E. coli* as well as avoid the phototoxicity of UV on normal tissue.^168^ The antibacterial activities of the CORMs were possibly related to the generation of ROS and the release of metal ions during treatment.

Similar to CORMs, metal-based nitric oxide-releasing molecules (NORMs) have also been discovered for the treatment of bacterial infections. The released nitric oxide (NO) can exert nitrosative and oxidative stress in bacterial cells. This strategy as well as other multimodal antibacterial therapy in combination with CORMs and NORMs has been recently reviewed extensively.^169^ Nevertheless, we anticipate that more attention shall be paid to elucidating the mechanisms of action of CORMs and NORMs in the future.

## Perspective and Outlook

We have summarized the potential of the metal-based strategy in the fight against AMR as well as how metallo-compounds could be utilized against bacterial infections via multiple approaches. Metallo-drugs offer several advantages over traditional organic small-molecule drugs, including high potency, unique mechanisms of action, and a broad range of targets, resulting in less likelihood to develop resistance. In addition to their inherent antimicrobial activities, metallo-antimicrobials also exhibit synergistic effects with certain antibiotics and thus can be used as antibiotic adjuvants to restore or enhance the efficacy of clinically used antibiotics, thereby prolonging their lifespan against various multi-drug-resistant bacterial strains.

The knowledge of the mechanism of action is the key to the development of more potent drugs. Extensive studies have revealed that most of the metallo-antimicrobials exert their antibacterial activities by releasing metal ions that bind to target proteins/amino acid residues or catalyze reactions with target proteins through changing their binding properties, which disrupts the functions, suppresses the protein expressions, interferes with the regulation of metabolism, or causes ROS damage in the bacterial cells. Such a unique mechanism of action makes it difficult for bacteria to develop resistance, thus making metallo-antimicrobials a promising weapon to tackle the AMR crisis. With the development of advanced technologies such as metallomics and metallo-proteomics, single-cell techniques, and artificial intelligence (AI),^170^ the mechanism of action of metallo-drugs will be further unveiled, allowing a deeper understanding of the mechanism of action of metallo-drugs in different dimensions,^171^ which provides fundamental guidance for the design and development of more potent metallo-antimicrobials.

Although metallo-antimicrobials are typically considered to be potentially toxic, not all metals possess intrinsic toxicity. The extent of toxicity depends on various factors, including the oxidation state of the metal ions, the ligand properties, and the delivery systems. The broad range of targets of metallo-antimicrobials can be a double-edged sword, as it may also lead to toxicity toward eukaryotic cells, thereby limiting their development and clinical use. Therefore, it is necessary to simultaneously evaluate their efficacy together with their pharmacokinetics and toxicity, which can be fine-tuned by the incorporation of different accompanying ligands, using different delivery systems or administration via different routes.^172,173^ A delicate balance is essential for advancing the field of antimicrobial drug development and addressing the growing concern of AMR. In addition, the combination of a clinically used (metallo)drug with antibiotics may readily overcome the shortage of potential toxicity of metal compounds for the treatment of infections by resistant bacteria.^115,174,175^ For example, an orally administered bismuth drug together with N-acetyl cysteine as an anticoronavirus cocktail therapy is currently in a phase-II/III clinical trial.^184^

Computational studies and machine learning models are promising and powerful tools for discovering metal–protein interactions and identifying potential targets for metal-based inhibitors. Metal complexes could be synthesized and characterized by computational methods including DFT (density functional theory), molecular docking, and target prediction to uncover the coordination behavior, biomolecular interaction, and possible protein binding sites.^2^ These basic statistical and theoretical investigations can lead to a deeper understanding of targets, dynamics, and ligand design. In addition, the use of artificial intelligence (e.g., AlphaFold) to track and/or predict potential protein targets of a metallo-drug has become a promising strategy.^176^ By employing sophisticated algorithms and simulations, we may predict the metal-binding site in proteins^171,177^ or related metal-binding site mutations.^170^ Using machine learning to screen and predict metallo-protein inhibitors is a more effective strategy compared with traditional molecular docking.^178,179^ The combination of AI-based methods with traditional inhibitor screening methods will enable more metal-based antimicrobial agents to be designed and synthesized. Moreover, the application of metallo-proteomics will allow more druggable targets to be discovered.^67^

Despite significant challenges, metallo-pentimicrobials offer a valuable perspective on the therapeutic intervention of infections caused by antimicrobial-resistant bacteria. With continued research and innovation, they hold the potential to be used either alone as antimicrobials or in conjunction with antibiotics to tackle AMR, improve patient outcomes, and address unmet medical needs.
